# The Public Health Exposome: A Population-Based, Exposure Science Approach to Health Disparities Research

**DOI:** 10.3390/ijerph111212866

**Published:** 2014-12-11

**Authors:** Paul D. Juarez, Patricia Matthews-Juarez, Darryl B. Hood, Wansoo Im, Robert S. Levine, Barbara J. Kilbourne, Michael A. Langston, Mohammad Z. Al-Hamdan, William L. Crosson, Maurice G. Estes, Sue M. Estes, Vincent K. Agboto, Paul Robinson, Sacoby Wilson, Maureen Y. Lichtveld

**Affiliations:** 1 Research Center on Health Disparities, Equity, and the Exposome, University of Tennessee Health Science Center, 66 N. Pauline, Memphis, TN 38105, USA; E-Mail: pmatthe3@uthsc.edu; 2 Department of Environmental Health Sciences, College of Public Health, Ohio State University, Columbus, OH 43210, USA; E-Mail: hood.188@osu.edu; 3 Vertices, Inc., 317 George Street 411, New Brunswick, NJ 08901, USA; E-Mail: wansooim@gmail.com; 4 Department of Family & Community Medicine, Meharry Medical College, Nashville, TN 37208, USA; E-Mails: rlevine@mmc.edu (R.S.L.); vagboto@mmc.edu (V.K.A.); 5 Department of Sociology, Tennessee State University, Nashville, TN 37209, USA; E-Mail: bkilbourne@tnstate.edu; 6 Department of Electrical Engineering and Computer Science, University of Tennessee, Knoxville, TN 37996, USA; E-Mail: langston@eecs.utk.edu; 7 National Space Science and Technology Center, Universities Space Research Association, NASA Marshall Space Flight Center, Huntsville, AL 35805, USA; E-Mails: mohammad.alhamdan@nasa.gov (M.Z.A.); bill.crosson@nasa.gov (W.L.C.); maury.estes@nsstc.uah.edu (M.G.E.); sue.m.estes@nasa.gov (S.M.E.); 8 National Space Science and Technology Center, University of Alabama, Huntsville, AL 35805, USA; E-Mail: maury.estes@nsstc.uah.edu; 9 Department of Ophthalmology, Charles R. Drew University of Medicine and Science, Los Angeles, CA 90059, USA; E-Mail: paulrobinson@cdrewu.edu; 10 Maryland Institute for Applied Environmental Health, School of Public Health, University of Maryland, College Park, MA 20742, USA; E-Mail: swilson2@umd.edu; 11 Department of Global Environmental Health Sciences, School of Public Health and Tropical Medicine, Tulane University, 1440 Canal Street, New Orleans, LA 70112, USA; E-Mail: mlichtve@tulane.edu

**Keywords:** exposome, public health, health disparities, trans-disciplinary, exposure science, social-ecological, combinatorial analysis, CBPR, geographical information systems, PPGIS

## Abstract

The lack of progress in reducing health disparities suggests that new approaches are needed if we are to achieve meaningful, equitable, and lasting reductions. Current scientific paradigms do not adequately capture the complexity of the relationships between environment, personal health and population level disparities. The public health exposome is presented as a universal exposure tracking framework for integrating complex relationships between exogenous and endogenous exposures across the lifespan from conception to death. It uses a social-ecological framework that builds on the exposome paradigm for conceptualizing how exogenous exposures “get under the skin”. The public health exposome approach has led our team to develop a taxonomy and bioinformatics infrastructure to integrate health outcomes data with thousands of sources of exogenous exposure, organized in four broad domains: natural, built, social, and policy environments. With the input of a transdisciplinary team, we have borrowed and applied the methods, tools and terms from various disciplines to measure the effects of environmental exposures on personal and population health outcomes and disparities, many of which may not manifest until many years later. As is customary with a paradigm shift, this approach has far reaching implications for research methods and design, analytics, community engagement strategies, and research training.

## 1. Introduction

Since the release of the Secretary’s Task Force Report on Black and Minority Health [1] the health disparities burden borne by racial/ethnic and other underserved populations has shown little improvement. Since 2003, Blacks have shown no significant change in disparities compared to Whites on 60 of the 73 measures of health care quality and access tracked and measured by the Agency for Healthcare Research and Quality [2], while for two, disparities actually increased (breast cancer diagnosed at advanced stages among women 40 and over, and maternal deaths per 1000 live births). It has become increasingly evident that without a radical new approach in efforts to comprehend the underlying causes of health disparities, our efforts to eliminate them are unlikely to succeed [3].

The causes of health disparities are varied, complex, and not yet well understood [4,5,6,7,8,9,10]. Only 10% to 30% of the variance in cancer and chronic disease outcomes, for instance, has beenattributed to genetic factors while the remaining 70%–90% has been attributed to the environment [11,12]. There is increasing evidence that social and ecological factors must be addressed together to eliminate health disparities [13,14,15,16].

Exposure science has evolved over the past thirty years as a distinct field, drawing from many disciplines to shed light on the effects that environmental exposures have on acute and chronic health conditions [17,18,19,20,21]. Exposure science is the study of stressors, receptors, and their sources of human contact within the environmental context of space (geographic location, e.g., latitude/longitude coordinates), place (attributes assigned to the location, e.g., home, work, park), and time [22,23,24,25,26,27]. The goal of exposure science is to identify and understand fundamental, shared mechanisms and common biological pathways (e.g., inflammation, methylation, oxidative stress, and other epigenetic changes) underlying a broad range of complex diseases. This has direct implications for the development of targeted personal and community health interventions [28,29,30,31,32].

The need for an exposure science paradigm shift was identified in the recent U.S. National Research Council publication: “Exposure Science in the 21st Century: A Vision and a Strategy” [17]. This report argued in favor of a broader approach to exposure science. In addition to a focus on human health, the report also recommended collecting exposure data that characterizes the ecosystem, recognizing that a healthy ecosystem is among the prerequisites for human health. It also suggested that adoption and validation of an exposomics context “should lead to the development of a universal exposure-tracking framework that allows for the creation of an exposure narrative, the prediction of biologically relevant human and ecologic exposures, and the generation of improved exposure response.” The lack of this “exposure narrative” significantly hampers community level exposure investigations, especially those associated with technological disasters such as the Gulf Oil Spill in 2010 [33].

Increasingly, investigators have adapted exposure science and social-ecological models to glean insights into the underlying causal mechanisms through which environmental exposures affect personal health which may lead to population level disparities [9,13,20,34,35,36,37,38]. Yet, traditional exposure science models typically have attempted to examine the impact of the environment on disease through a reductionist approach, supported by discipline-driven, theories that have led to narrowly focused assessments, models, and analytics [11,17].

The exposome has been proposed as an emergent exposure science paradigm for conceptualizing the cumulative effects of environmental exposures across the lifecycle (from conception to death) and for examining the dynamic, multi-dimensional inter-relationships between environment and health [3,20,21,23,24,25]. In 2005, Wild [22] first described the exposome—the totality of one’s lifetime exposures—as a conceptual framework for understanding the environmental context of health outcomes. He differentiated between the “eco-exposome” as the point of contact between an external environmental stressor and biological receptor of an individual and the “endo-exposome” as the inward effects arising from exposure on those receptors. A related term, the envirome, was initially used to describe triggers affecting psychiatric illness [39]. Increasingly, in the context of environmental health the envirome represents the constellation of psychosocial factors that influence health. The term has also been used to describe the effect of the environment on genotype, resulting in phenotypic variability [40]. To date, source-exposure-disease relationships have been addressed primarily at an endogenous level, with a focus on the effects of exposure inward on human, internal chemical environment [29]. Characterization of the “eco-exposome” has been far more challenging [22].

In 2011, we received a supplemental award from the Environmental Protection Agency to our P20, NIMHD Health Disparities Research Center of Excellence at Meharry Medical College to establish an Environmental Health Core (Grant Number 3P20MD000516-07S1). The aims of this grant were to: (1) establish a core that supports analysis of the complex interactions between health outcomes, disparities and the environment; (2) promote the use of trans-disciplinary models and analyses to increase knowledge about the complex relationships between health disparities and the environment, and (3) use public participatory geographic information systems (PPGIS) to engage community stakeholders in the use of spatial data and interactive mapping to reduce health disparities. To achieve these aims, the Center engaged a transdisciplinary group of investigators representing a broad array of disciplines and skills, including: (1) the physical, built, social and policy environments; (2) health disparities content areas; (3) a range of statistical models and analytic techniques including multi-level analysis, predictive modeling, spatial analysis, and graph theory/combinatorial analysis to analyze the complex relationships between health disparities and environmental factors; (4) bioinformatics, and (5) decision support tools for engaging community members in the research process, including data collection. This new approach has implications not only for how we conceptualize health disparities and conduct health disparities research, but also for strategies to improve health outcomes and eliminate disparities in vulnerable subpopulations. A glossary of trans-disciplinary terms is presented in Appendix 1.

The objectives of this manuscript are to:

(1) present the public health exposome as an integrated model for examining exogenous and endogenous source-exposure-disease relationships across the life cycle and the influence of those relationships on health disparities at a population level;
(2) describe the public health exposome database, a 30-year, longitudinal repository that integrates health and environmental databases;
(3) provide an overview of the transdisciplinary methods and analytics we have developed to help unravel the complex interactions between environmental stressors and bio-psycho-social systems at the individual, community, and social-ecological systems levels, as those relate to personal health and population level disparities;
(4) discuss the use of emergent sources of exposure data and the interface with bioinformatics and community engagement; and
(5) examine the implications of the public health exposome paradigm for future health disparities research.

## 2. Public Health Exposome

### 2.1. Concepts

A public health exposome paradigm is grounded in systems theory [41] and a life cycle approach [42]. It provides a conceptual framework which can be used to identify and compare relationships between differential levels of exposure at critical life stages, personal health outcomes, and health disparities at a population level across space, place, and time. It allows for the generation and testing of hypotheses about the pathways through which exogenous and endogenous exposures result in poor personal health outcomes and population level health disparities and enables the identification of at-risk persons and health disparities communities and the targeting of public health interventions.

**Figure 1:**
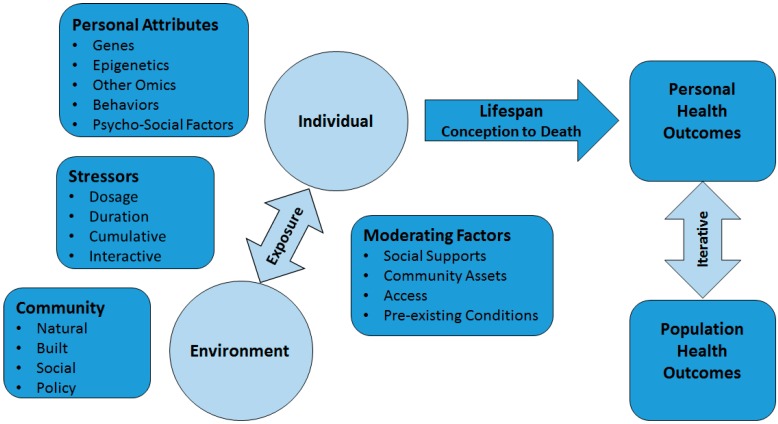
Public health exposome conceptual model.

In contrast to previous descriptions of the “exposome” which have focused on the effects of endogenous exposures, a PHE approach integrates information about endogenous and exogenous exposure mechanisms, processes and outcomes with mediating and moderating factors at both the individual and population health levels. The below PHE conceptual model (See Figure 1) presents an integrative approach that can be used to both generate and test hypotheses about the underlying causal mechanisms through which environmental exposures act at critical life stages from the molecular to a population health level. This model has guided the development of a bioinformatics infrastructure that supports the assessment of exposure characteristics on personal health outcomes across the lifespan and challenges us to reexamine our understanding of the relationship between personal health and population level, health disparities.

### 2.2. Data Sources

An exposure science approach has guided the development of the PHE conceptual model, data repository, and bioinformatics infrastructure by this transdisciplinary team of inter-institutional investigators which convened over the past three years through weekly teleconferences and quarterly in-person meetings. This PHE conceptual model has guided formative research efforts for conceptualizing pathways and generating hypotheses through which environmental exposures lead to population level health disparities and the establishment of a data repository for analyzing the direct and indirect effects of environmental exposures across space, over time, and across the life span [34].

The PHE data repository includes data captured at various spatial and temporal units. It currently stores over 12,000 variables that have been geocoded and are being harmonized at an annual and county level in a relational database. Most of the health and environmental data in the PHE data repository were obtained from publicly available sites at no or low costs; many were downloaded directly from the web. However, some restricted data also are included in the data base. Approval was received from the Meharry Medical College IRB to cover handling and access to restricted data to ensure confidentiality. A comprehensive list of data sources of the data we have collected in the public health exposome repository is provided in Appendix 2.

The goal of the PHE repository was to collect annual, health and environmental exposure data, for 3141 counties and county equivalents, over 30 years (1980–2010). While we recognize the importance of using more granular levels of spatial and temporal data, such as block groups or neighborhoods, apart from data provided by the U.S. Census, these data are rarely publically available. The data repository currently includes both raster (e.g., area level) including remotely sensed meteorologic data [43,44,45,46] and vector (point) data and various shape files (e.g., census tracts, zip codes, metropolitan statistical areas (MSAs), *etc.*). The database also includes crosswalks which allow us to convert spatial units to other units. Crosswalks allow for data obtained in smaller geographic units such as census tracts, grid cells, or zip codes to be aggregated up to the county level. Health and some exposure data, however, often are only available at larger spatial units (such as (MSAs), hospital referral regions (HRRs), and hospital service areas (HSAs) and as such, typically are more challenging to convert to counties or county equivalents. We are exploring the use of visualization, imputations, and synthetic estimates to apply attributes of the population from a larger unit to that of a smaller unit and other methodical approaches for characterizing uncertainty due to exposure misclassification, multiple sources of data, and different spatial scales [47,48].

A taxonomy for distinguishing and measuring the effects of different types of health and exogenous exposures included in the database was developed. Health data were organized around six broad areas of disparities (cancer, cardio-metabolic disease, HIV/STIs, intentional/unintentional injury, maternal and child health, and mental health and substance abuse) [1], and include measures of mortality, morbidity, screening and behaviors. Environmental data are organized in four broad domains: natural, built, social and policy environments. The natural environment includes exposure measures of air, climate, water, and land; the built environment includes attributes of places we live, work, play, learn and pray, with measures of both quality, quantity, and access; the social environment includes descriptors of social/economic conditions such as poverty, crime, racial segregation, and unemployment found in an area or population; while the policy environment includes data about governmental laws, ordinances and regulations that have either a direct or indirect impact on health.

Data were originally collected and stored in ArcView 10.2, geographic information system (GIS) and later reprocessed as a relational database in Microsoft SQL Server to support data queries and the exporting of data to statistical software packages, including SPSS, SAS, R and STATA, and for use by computational biology colleagues to perform high throughput and combinatorial analysis. Having data in a relational database allows for easier data queries in a format that does not require knowledge of a GIS software program. A comprehensive data dictionary with metadata for all data elements has been created in MS Access. We expect that the PHE database will continue to grow as more data become available over time and to be continually refined as spatial-temporal and computational modeling and analytic tools are developed.

## 3. Analytic Approaches

A PHE approach supports the application of both parametric and non-parametric methods and statistical analyses, including those that are both data and hypothesis driven and is not limited to underlying assumptions of independence or normal distribution. The PHE data infrastructure supports the handling of “Big Data”, non-parametric probability distributions, and spatial and temporal auto-correlation and can be used by a broad array of research approaches and analytics, including Bayesian models, predictive modeling, computational spatial analysis, and graph theory/combinatorial analysis and simulation—analytic tools used more commonly in other disciplines, such as mathematics, GIS, engineering, and computational biology. Three approaches that take advantage of the relational structure of the database show great promise for future use with the public health exposome include multi-level, spatial, and computational and combinatorial models and analytics. A comparison of the three approaches is presented in Appendix 3 (Table A1).

### 3.1. Multi-Level Analysis

A multi-level approach that explicitly recognizes the embedded nature of health outcomes within its biological, social, ecological, and community contexts is likely to provide a better understanding of disease across the life course and to explain heterogeneities in health disparities across socioeconomic and geographic boundaries that, to date, remain largely unexplained. [49,50]. This approach underscores the need to measure health disparities within a social-ecological context as opposed to examining health disparities without any reference to environmental characteristics [51,52].

Multi-level analyses are particularly suitable for analyzing contextual data, because they take into account the hierarchical structure and the nested nature (spatial and temporal) of the database, allowing for the simultaneous examination of individual and group-level factors. Other advantages of multi-level analysis include being able to differentiate “independent” effects, assess the reciprocal relationships between factors at different levels, and estimate how much complex between-group variability is explained by contextual factors [37,52].

**Figure 2:**
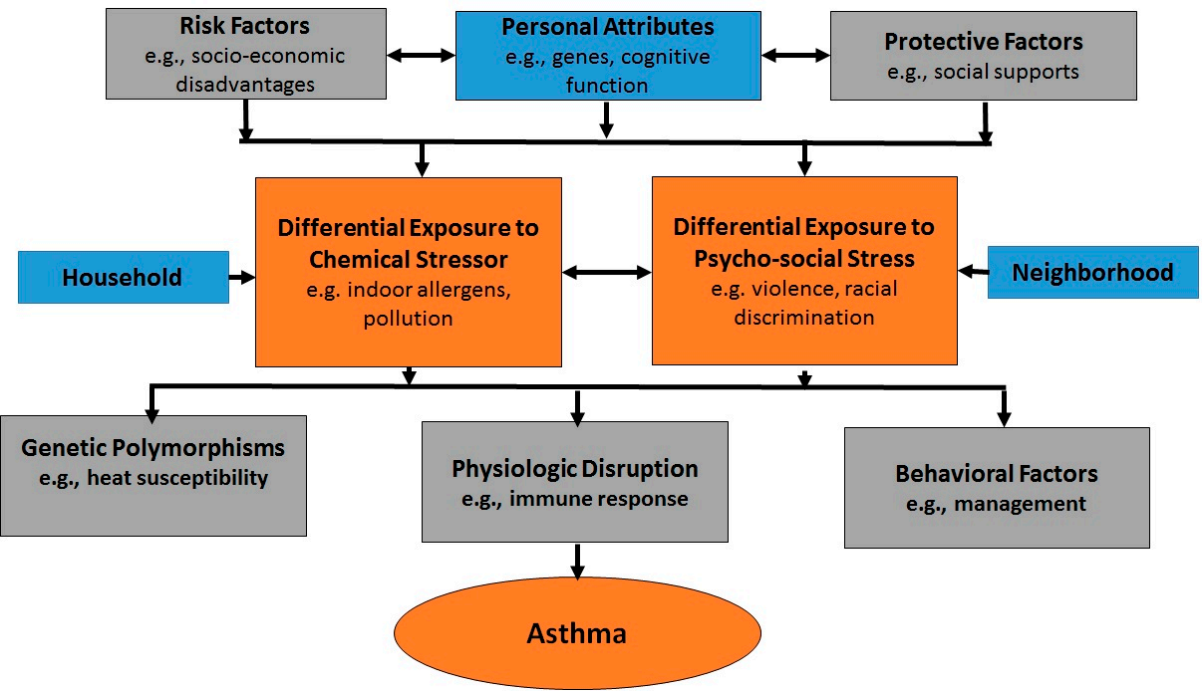
A multi-level ecological approach to explain heterogeneities in asthma expression across socioeconomic and geographic boundaries.

For example, variations in asthma occur within a social and ecological context that can benefit from employing an explicit multi-level analytical strategy (see Figure 2). Such an approach allows the investigator to: (1) quantify the extent to which asthma is clustered by neighborhood and community grouping; (2) quantify both the extent to which neighborhood variations in asthma are due to the clustering of risk factors and the extent to which the effect of individual risk factors vary from neighborhood to neighborhood; and (3) quantify the relative importance of individual, neighborhood and societal level exposures in predicting individual asthma. These three constitutive components of a multi-level analytic framework have important implications for asthma as well as other areas of disparities research.

### 3.2. Spatial-Temporal Analysis

GIS and spatial-temporal analysis also offer useful tools that can be used both for data visualization of spatial and temporal patterns [53,54] and for analysis of spatially and temporally continuous data. Mapping can be used to visualize geographic patterns and temporal trends at a county level, generate hypotheses, and identify “hot spots” to guide further data collection efforts and the targeting of public health interventions. In addition to generating traditional static maps (e.g., rates per 100,000), GIS supports data visualization tools for modeling spatial and temporal relationships. Spatial analysis provides the tools to examine the relationships between socioeconomic riskscapes, environmental health hazard sources, socially vulnerable neighborhoods, and health disparities, including hotspot analysis and cluster and outlier analysis to display changes in patterns over time. Spatial statistical methods such as kernel density estimation, trend surface analysis, seasonal time series trend decomposition, and spatial autocorrelation are geo-statistics that can be used for analyzing the spatial and temporal relationships that exist among diseases, environments, population characteristics and health disparities within or between defined populations and geographic areas.

In recent years, there have been increasing efforts to use GIS and mapping to visualize spatial and temporal aspects of public and environmental health issues. The PHE approach differs in a number of important ways from other public health data sharing and visualization portals that have been developed, such as those supported by the Center for Disease Control and Prevention’s National Environmental Public Health Tracking Network (http://ephtracking.cdc.gov/showHome.action) [55], the Environmental Protection Agency’s EJView (http://epamap14.epa.gov/ejmap/entry.html) [56], Community Commons (http://www.communitycommons.org/maps-data/) [57], Opportunity Nation (http://opportunityindex.org) [58], and the Healthy Communities Institute (http://www.healthycommunitiesinstitute.com/) [59]. First, the PHE approach provides a comprehensive and integrative theoretical framework for identifying environmental exposures and for conceptualizing the mechanisms through which exposures affect biological processes, personal health outcomes, and lead to population level disparities. An underlying theoretical model is generally lacking in these other approaches. Second, the PHE is not limited by traditional disciplinary boundaries in conceptualizing exposures (e.g., public health, environmental health, social determinants, or public policy) which underlie these other efforts. Third, the PHE is more than a mapping tool; it is fundamentally a research tool. While we recognize the importance of visualization and community mapping—especially as web tools—online efforts are limited by their ability to display multiple types of vector and raster data simultaneously; it quickly overwhelms. Fourth, detecting patterns from maps often is a matter of spatial and temporal scale which may be extremely difficult to capture on a web portal. Lastly, the PHE repository supports multiple types of tools and analytics which can be used to address limitations that arise from using geo-spatial data, including spatial autocorrelation and spatial uncertainty.

### 3.3. Combinatorial Analysis

“Big Data” analytics is the process of examining large amounts of data to detect hidden patterns, unknown correlations and other useful information. In this paper, we use the term “Big Data” to refer to the use of data driven approaches, such as graph theory and combinatorial analysis, rather than referring to the sheer size of the data [60].

The application of “Big Data” analytics and tools to the public health exposome data can be used to help explain heterogeneities in health disparities across socioeconomic and geographic boundaries that to date, have remained largely unexplained. Combinatorial analysis employs high performance parallel implementations using the power of mathematical abstraction to compile correlations in “Big Data” sets into statistically robust inter-related clusters. By extracting and highlighting variable sets common to multiple relationships (cliques and other dense sub-graphs), these tools can be used to determine inflection points and other patterns of possible interest, and to perform analysis using the latest mathematical tools, innovative graph algorithms, and powerful computational platforms to uncover latent but meaningful relationships on an immense scale [60]. Methods for querying and mining “Big Data” are fundamentally different from traditional statistical analysis on smaller samples. “Big Data” often is more valuable than small samples because general statistics obtained from frequent patterns and correlation analysis usually overpower individual fluctuations and can disclose more reliable hidden patterns and knowledge. Further, interconnected “Big Data” forms large heterogeneous information networks with which information redundancy can be explored to compensate for missing data, crosscheck conflicting cases, validate trustworthy relationships, disclose inherent clusters, and uncover hidden relationships and models. Most clustering approaches are limited by the fact that the clusters produced are disjointed, requiring that a variable be assigned to a single cluster. This greatly simplifies the analysis. One challenge is to define these filters in such a way that they do not discard useful information.

By employing high performance parallel implementations and the power of mathematical abstraction we have been able to extract and highlight variable sets common to multiple relationships and to determine inflection points and other patterns of possible interest. High throughput analysis (HTA) uses the latest mathematical tools, innovative graph algorithms, and powerful computational platforms to uncover latent but meaningful relationships on an immense scale, providing research opportunities to data mine at multiple levels of granularity. We have used HTA (combinatorics and graph theory) to compile weak correlations using a subset of 600 variables from more than 12,000 PHE variables into statistically robust inter-related clusters and to identify relevance networks, beginning with a symmetric correlation matrix [60].

### 3.4. Multi-Modal Analytic Approach

We have found it advantageous to apply multiple types of analytics to address the complex relationships between environmental exposures and health disparities. For example, while many individual risk factors associated with poor pregnancy-related outcomes are known, limited progress has been made to date regarding the complex spatial-temporal nature of the relationships between pregnancy related morbidities and mortalities, environmental exposures and population level disparities. The below example illustrates the use of a multi-model analytic approach to examine these complex relationships across states combining the use of GIS and spatial analysis, graph theoretical analysis, combinatorial analysis, and traditional statistical analytics.

We used a PHE approach to examine the social-ecological context of premature birth [61]. GIS was used to map the rates of premature birth at a county level for the 50 states. A county-level database was created to integrate health outcomes and environmental exposure data from each of the four PHE domains (natural, built, social and policy environments). Graph theoretical algorithms, combinatorial analytics and traditional statistical methods then were used to uncover putative inter-linking associations of PHE exposure variables (natural, built, social and policy environments) with premature birth rates. For a more detailed discussion of these methods, see Langston, *et al.* [60]. Using county-level data from the PHE database, we first constructed a complete, finite, simple, undirected and edge-weighted graph, where each node represented a variable, and each edge was weighted by the correlation coefficient of its endpoints. Paracliques/factors were derived from 600 exposure variables using combinatorial analysis. Spatially adjusted regression analysis, with and without spatial autocorrelation, was then used to model pregnancy related outcomes. This approach allowed inclusion of a large number of different and highly divergent population level variables, reducing the number of variables under review through graph theoretical techniques, which permitted us to apply traditional and otherwise unscalable statistical analysis techniques. Resulting models explained a substantial proportion of pregnancy related health outcomes supporting the value of the PHE approach in studying health disparities [61].

## 4. Implications

**Figure 3:**
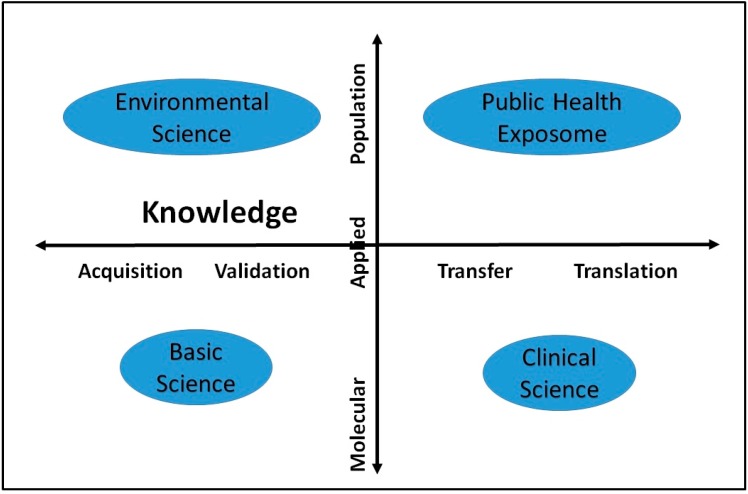
Application of the public health exposome in environmental health research.

The PHE paradigm provides opportunities to strengthen the evidence base for health disparities research, practice, policy, community engagement, and research training in unique, transdisciplinary, and interdependent ways, supporting research advances in the following areas: individual exposure characterization; community-level, environmental, epidemiologic cohort studies; health disparities research; community-based participatory research (CBPR) methods; research at the intersection of the eco-system and human health; and training a new cadre of emerging transdisciplinary scholars. Figure 3 presents two research trajectories: research targeting knowledge gain as depicted on the X-axis represents a continuum from knowledge acquisition, to validation, transfer and ultimately to translation. On the Y-axis, research occurs along the translation continuum from molecular (basic) to applied and ultimately to population-level inquiries. A significant amount of research in general, and exposure research specifically, has been conducted in the basic sciences to achieve knowledge acquisition or validation (left quadrants) of the effects of environmental exposures. A growing, but still limited body of work is devoted to addressing health disparities at the population level and translation beyond the traditional clinical settings (right quadrants).

### 4.1. Research Implications

For environmental health research, population level studies (right upper quadrant of Figure 3) traditionally have been hampered by limitations (small denominator, incomplete exposure characterization) that potentially now can be addressed by a PHE approach. Specifically, the vast database harnessed by the PHE can allow for enhanced risk assessments using reasonably acceptable surrogates, as well as facilitate health outcome analyses using a robust denominator. In turn, the possibility of doing both types of assessments informs the design of tailored environmental epidemiologic studies, increasing the likelihood for meaningful results in a resource- effective manner.

**Figure 4:**
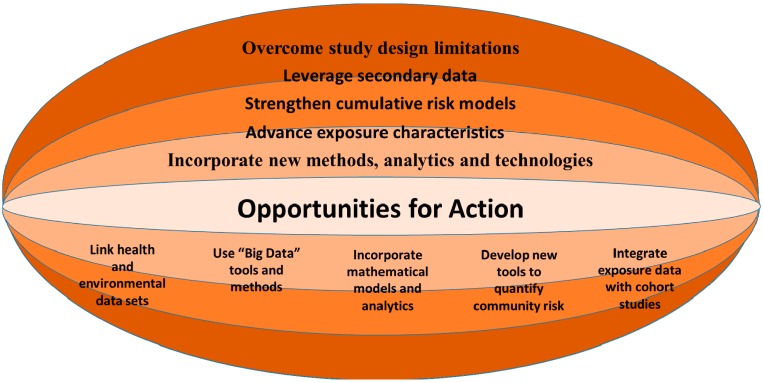
Public health exposome: advancing health disparities research.

The PHE advances health disparities research in five critical aspects (see Figure 4): (1) *Overcomes study design limitations.* Human health studies favor a randomized controlled trial design, the gold standard in providing the most direct evidence of causation. This design is frequently not possible to implement because exposures to contaminants have often already occurred and prospectively exposing humans is ethically inappropriate. Obtaining a representative study sample and control subjects, and confounding social determinants are among the most common limitations of environmental studies. Several of these limitations are now addressed by deploying the PHE paradigm. (2) *Leverage secondary data:* Specifically in the context of environmental health, data not originally collected to assess potential exposures to hazardous substances are seldom useful even as baseline measures. The PHE database and analytics suite will, at a minimum, enable geospatial documentation of contaminant sources to target potential populations at risk for further evaluation taking into account the requirement to assess the presence of potentially completed exposure pathways. (3) *Strengthen cumu lative risk models*. By taking a life course approach as well as integrating multiple proximal and distal factors influencing health disparities, public health research can target primary data collection more effectively time- and cost-wise. In addition, the PHE can support the development of predictive models, facilitating tailored prevention and intervention efforts in vulnerable communities. (4) *Advance exposure characterization.* Insufficient exposure characterization, especially past exposures, and lag time between exposure and study significantly hamper the conduct of environmental epidemiologic studies. The PHE framework offers important opportunities to strengthen the knowledgebase of exposure science. For example, the framework operationalizes environmental exposures in four broad domains: natural, built, social and policy. The PHE enables capture of nested complexities of exposures, and provides a model that allows for the integration of exposure data on personal, bio-psycho-social- and health conditions taking into account the social-ecological context. Traditionally, the epidemiology of health disparities has focused on individual-level risk factors and family factors while far less attention has been given to the broader social-ecological context in which individuals live, work, learn, play, and pray. A more comprehensive approach that considers the range of social and ecological factors that co-vary with lower SES and minority group status (e.g., differential environmental exposures, residential segregation, psychological stress, housing quality, and social capital, *etc.*) is needed to tease apart these relationships [62,63]. (5) *Incorporates new statistical methods and technologies*. The PHE platform supports the collection of data in both a GIS and a relational database and provides multiple methods of data mapping and visualization and spatial, mathematical, and statistical modeling and analyses. These methods enable us to include many more variables than are usually included in environmental public health studies and to take advantage of the range of data that is now available.

### 4.2. Implications for Public Health Practice

The notion that the health of the environment is intimately linked to that of humans is receiving increased recognition [25]. A PHE paradigm puts us in a better position to address the role that historical burdens of exposures have manifested in all four PHE domains. The use of GIS and other spatial modeling and simulation tools can help research and community partners better visualize past environmental media contamination and prospectively model potential future risk [64]. Visualizing environmental contamination in this way demystifies exposure science and makes the limitations scientists face more real for community partners. The need to bolster the translation of ecosystem-human health research portfolio and tools by putting data into the hands of end users is becoming more urgent in the context of both natural and technological disasters such as the Hurricane Katrina, Super Storm Sandy, and the Gulf of Mexico oil spill [32]. Remote-sensing derived data and spatial analyses of both environment and human health data can significantly contribute both to this emerging research area as well as to public health practice and policy. For example, the use of remote sensing data allows us to identify areas affected by high levels of PM_2.5_ and to translate findings into tailored proactive health education messages targeting sensitive sub-populations such as children with childhood asthma as well as their health care providers. Employing the analytical capabilities of a PHE approach can be used to help us identify both distal and proximal surrogate indicators of exposure in the absence of direct exposure measurements.

In addition, a PHE approach has implications not only for health care, public and environmental health researchers but for health care, public and environmental health, and social service providers, as well. Tools such as health impact assessments (HIA), community mapping, and public participatory geographic information systems (PPGIS) can be used to facilitate not only collaborative learning but also to stimulate concrete and effective contributions to research, practice and policy beyond a specific study [65,66,67]. HIAs have proven to be informative, yet such assessments are not uniformly used when developing new environmental policies. A PHE database can complement HIAs by providing access to more accurate exposure data from a practice-based, upstream, historical context. Application of a PHE paradigm to health disparities provides the foundation for more effectively translating science into practice. Similarly, PHE derived data and maps can be used to assist health care providers make prescriptive recommendations about behaviors outside the clinic walls by identifying threats within the social ecological environments in which individual patients live, work, learn, play, and pray and resources to mitigate them.

### 4.3. Policy Implications.

From an environmental health perspective, more accurate exposure information at the individual and community level can facilitate translation into more effective health and environmental policies [68]. Likewise, gaps in existing policy can inform research priorities. For example, identification of vulnerable populations can lead to environmental health policies that offer better community protection, with an aim of promoting health equity in a proactive fashion rather than addressing environmental justice issues after the fact [68,69,70,71,72,73,74]. Informed by a PHE paradigm, science-driven environmental health policies then can be translated into evidence-based, frontline, public health practice to provide more effective, tailored health services for vulnerable sub-populations.

The analytical and technological functions supported by the PHE database also can serve both an important role in informing the development of new social and environmental policies and in evaluating the “prevention and protection effectiveness” of existing policies, to ensure they are not having the unintended consequence of increasing disparities. This is critical in light of work of Levine and colleagues [75,76,77] who have previously shown clear relationships between the passage of federal laws which were intended to increase coverage of health benefits, and subsequent increases in racial health disparities.

**Figure 5:**
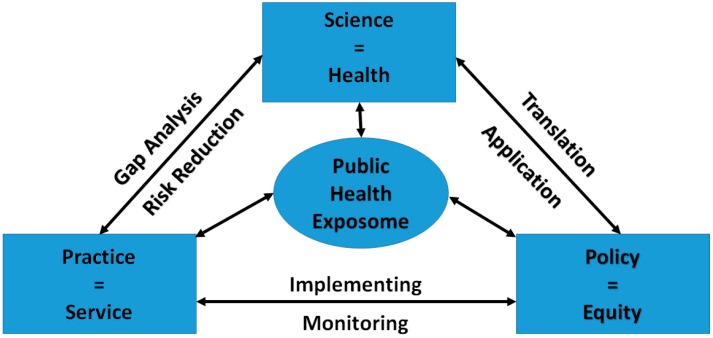
Public health exposome: informing science, policy and practice.

The PHE paradigm can be used to strengthen the linkages between science, practice and public policy, resulting in improved health and wellbeing (see Figure 5). PHE informed research should lead to the identification of gaps in practice and strategies for reducing risk. As with practice gaps, a PHE informed assessment of health system functioning can elucidate gaps in public policy, and strategies for translating and applying research findings into policy. Frontline public health practitioners are well positioned to inform the health disparities research agenda by providing real world feedback on the effectiveness of practice and policies, especially those originally intended to advance community protection and address historic burden of health disparities.

### 4.4. Community Engagement

Increasingly, community based participatory research (CBPR) methods have been employed to examine the social-ecological context of health disparities which considers the determinants of health and disease to include a broad range of factors, including biomedical, social, economic, cultural, historical, and political [78,79,80]. CBPR has been promoted as a co-learning and empowering process that facilitates the reciprocal transfer of knowledge, skills, capacity, and power between academic and community partners rather than being a specific research methodology [81,82,83]. There is increasing evidence which supports the use of CBPR as an effective strategy for building trust between researchers, communities and populations being studied, contributing to the quality of study designs, methods and dissemination of findings, and stimulating practice-based research. Because the research is “grounded” in the real life experiences of people, findings are more likely to lead to collective action and policy change [84]. Community-academic partnerships have proven specifically effective in post-disaster settings where the relevance of research to policy and practice is paramount [85].

*Public participatory geographic information systems* (PPGIS) offers a new approach for engaging community partners in health disparities research within a place-based ecological framework, advancing CBPR methods by employing crowdsourcing, smart phones, and other personalized data collection technologies and applications [86,87,88,89,90,91]. PPGIS harnesses emerging information tools and technologies to gather, measure, analyze, and map place-based, geospatial captured data which can be used to visualize population level risk factors and community assets, conduct environmental health impact and neighborhood needs assessments, and develop and implement targeted interventions aimed at improving health conditions at a neighborhood level [92,93]. PPGIS is a promising approach that can be used to provide: (1) community partners with access to historical data, resources, and interactive mapping tools that can be used to analyze the environmental context of user generated questions about health disparities; (2) baseline data which can be used to evaluate the impact of targeted interventions; and (3) an informatics infrastructure that can accommodate data collected from smart phone, crowdsourcing, and social media technologies. It also can help overcome challenges arising from limited publicly available data.

Our team has combined the use of PPGIS and crowdsourcing methods, customized smart phones, and interactive mapping websites to put the power of GIS into the hands of community members allowing community end users to visualize environmental conditions, assets, and risk factors at a neighborhood level with minimal training [34]. We currently are pioneering the integration of PPGIS methods with interactive mapping websites (see: http://www.immemphis.com [53], http://www.communitymappingforhealthequity.org [54] and which incorporate *Mappler*^©^ technology to provide community partners with access to data, maps, and decision support tools, enabling them to identify and address issues of concern without the need for academic intermediaries. *Mappler*^©^ is a smart phone application that supports real-time data collection at a neighborhood level by community partners [92] and is capable of taking full advantage of built-in, smart phone features, including camera, internet access, time stamp, light measures, gyroscope, accelerometer, GPS and Bluetooth technologies with near real time interface to internet linked databases.

In 2012, we used PPGIS and crowdsourcing methods and *Mappler*^©^ technology to address the gas shortages in the aftermath of Super storm Sandy in the New York/New Jersey region, in near real time [94]. *Mappler*^©^ was used to develop a customized brief survey for the smartphone which, in turn, was used by a group of volunteer high school students to call all of the service stations in the New York/New Jersey coast areas affected by Super storm Sandy about their gas availability [95]. Data were updated daily to the customized website: http://mappler.net/gasstation/ until the gas availability crisis subsided [96]. Survey questions included whether there was a telephone response, whether the gas station was open, their hours, if the service station had gas available, the types of gas availability, how long they expected it to last, current waiting time, and whether they were using a back-up generator for power. While the customized website was developed to respond to the gas availability needs of affected residents, *Mappler*^©^ also was used by both FEMA and White House officials during the immediate aftermath of Hurricane Sandy to monitor gas availability and plan emergency response efforts, demonstrating the huge upside potential of PPGIS and related crowdsourcing and social media technologies.

### 4.5. Transdisciplinary Research Training

Operationalization of a public health exposome into four broad domains has had the effect of stimulating transdisciplinary collaborations. This has profound implications both for how we conceptualize the composition of research teams as well as for how we organize and structure research training cores within health disparities and environmental health research centers. To effectively conduct research informed by a PHE approach requires research teams to include scientists with highly specialized knowledge and skills from a broad array of disciplines, including the natural/earth sciences, engineering and urban planning, social and behavioral sciences, and policy sciences; content experts in each of the broad areas of health disparities, including clinicians, toxicologists, and molecular epidemiologists; and highly specialized professionals, in mathematics and computer science, GIS, web design and information technology, bio-informatics, “Big Data”, and statistics. Creating a cadre of emerging, transdisciplinary scholars skilled in this broad array of fields however, will require the breaking down of barriers created by traditional academic disciplines as well as existing research funding mechanisms.

## 5. Limitations

Construction of a single, integrated, public health exposome database offers many challenges that will require significant commitment and resources to maintain and update. Obtaining data in each of the different domains of the PHE, for instance, provides its own set of unique issues. Some of these challenges are cross-cutting, while others are more specific to a specific domain or unique to a dataset.

Cross-cutting issues include a lack of standardization of how social constructs are defined and how variables are coded, spatially and temporally, data collection protocols, harmonization standards, policies, and regulatory frameworks. Development of a common informatics infrastructure that supports de-identification of data, data sharing, management, storage; protocols for updating data, standardization of data dictionaries including nomenclature and meta-data; and the use of restricted and crowd-sourced data also are needed. In addition, validation and vetting of data generated from social media have not yet been fully addressed.

Restrictions in accessing sub-county level health data present another challenge. These current restrictions prevent use of personal health data which might be useful in identifying and targeting “hot spots” and severely restrict research efforts that promote a population health approach to the elimination of health disparities. Laws that were enacted to ensure patient and/or provider confidentiality present a major challenge to the PHE approach and public health research. The Health Insurance Portability and Accountability Act of 1996 [97] and the Public Health Service Act (42 U.S. Code 242m(d)) [98] currently prohibit data collected by the National Center for Health Statistics from being displayed in any way that might allow the identification of an individual in a geographic area. However, there is no single standard used by federal, state and local agencies to protect confidentiality. Some agencies use a minimum numerator (e.g., HRSA data warehouse suppresses data for birth and infant mortality statistics for aggregated values where the value for any single combination of gender and race for the year is less than 10 for the given geography (http://datawarehouse.hrsa.gov/data/aboutdata/infantmortalitydatasupression.aspx) [99] while others use a minimum denominator (HIPAA guidelines allow record-level data to be shared for geographic areas with more than 20,000 people). Further complicating this is that policies on data suppression have changed over time based on disease and changes in laws resulting in different data use policies.

Another challenge we encountered was that annual, county-level mortality data from CDC Wonder (the main source for mortality data) often are unavailable due to small numbers resulting in a large amount of unreliable data, particularly in rural counties, where the number of annual, cause specific deaths are fewer than ten. To get around this, we aggregated multiple years of data to be able to generate a rate for a multi-year period. Without access to geo-spatial information for sub-county health data, future opportunities to address population health and target interventions to health disparities populations in greatest need will be severely hampered and its usefulness for identifying and targeting disparities limited.

One of the greatest challenges in adapting a PHE approach, however, is that it will require a revolution both in the ways that science is conducted and funded [100]. The PHE moves away from a traditional, categorical, reductionist approach towards an integrated, holistic approach in understanding the causal pathways through which exogenous and endogenous exposures operate, their impact on biological and bio-psycho-social systems, and ultimately on their combined impact on personal health and community-level health disparities. This falls outside the current domain of any specific NIH center or institute raising the question of which agency will fund this type of transdisciplinary research.

## 6. Conclusions

Scientific understanding of how external exogenous environmental exposures are related to individual endogenous exposures at the target organ level and the effects of these exposures on biological systems and personal health outcomes and their potential impact on population health disparities is in its formative stages. A PHE paradigm envisions the origins of health disparities as the continuous and dynamic interface between person and environment from conception to death, over time, space, and place. It uses a life course, systems approach and a social ecological theoretical framework for linking exogenous and endogenous exposures across the lifespan, providing more complete exposure pathway models, and engaging community participation in the research process. The PHE provides opportunities for compiling a more complete exposure profile than what is available today in terms of space, place, and time with more accurate measures of when, how often, how long, and how much exposure occurred. It adds to the current exposomics literature by increasing our understanding of the impact of multiple exogenous exposures, the social gradient of health, how biological systems are affected over time by environmental exposures, the impact of those exposures at a molecular level, and their combined contribution to disparities at a population level. The speed with which our understanding of the relationships between exogenous and endogenous exposures develops will have a great deal to do with the capacity of PHE data to achieve its full potential.

The current PHE data repository was derived primarily from publically available datasets. The data infrastructure allows both for ongoing expansion of the database, including the addition of new data sets and additional years of data, as well as for the incorporation of greater amounts of both personal and population level data from a variety of sources, such as local government, electronic health records, wearable health devices, PPGIS crowd sourcing, and web mapping services (WMS). With adequate safeguards, the data infrastructure could readily be expanded to include personal health data, such as those derived from electronic health records, longitudinal cohort studies, syndromic surveillance systems, and health information exchanges.

A PHE approach supports retrospective and prospective systems theory modeling and methods, including advanced and complex multi-level, spatial, Bayesian, and high throughput mathematical designs. Towards this end, we have pioneered the use of data-driven, graph theory/combinatorial techniques and analytics from computational biology to identify relationships among the myriad of environmental exposure and population health data points. High throughput mathematical analysis strategies allow for analysis of vast amounts of secondary data which allow for a larger study denominator, overcoming a major flaw of many environmental epidemiologic studies. The spatial, temporal, and nested nature of the dataset makes it a natural source for applying a wide range of analytics including Bayesian, multi-level, and computational spatial analysis.

The use of longitudinal data also provides an alternative approach to collecting cost-prohibitive exposure data. Used in conjunction with longitudinal cohort studies that incorporate biological, genomic, and tissue samples with clinical and social-behavioral data, the PHE approach has the potential to move science a step closer towards identifying the biological mechanisms through which environmental exposures affect health and how they result in population level health disparate.

The most significant contributions of the PHE are in strengthening exposure characterization from three distinct but interdependent perspectives: data generation, storage, and analysis. Other scientific contributions of a PHE include how we conceptualize entire, exposure-disease pathways, the need for transdisciplinary research teams and training programs, and how we adopt new methods that utilize data generated from rapidly developing technological changes in biomedical informatics (electronic health records, personal health monitoring devices), GIS, crowdsourcing, smart phone, computer, and web technologies.

A paradigm shift to a PHE approach also provides a promising roadmap for engaging community partners and for creating a stronger, multi-faceted evidence base for environmental justice. Engaging community partners in research has long been recognized as important not only for the development of a better understanding of the causes of health disparities but for the design of more effective community interventions and environmental policies. The addition of PPGIS tools such as *Mappler*^©^ interactive mapping technology significantly advances opportunities for community engagement by providing community partners with access to technologies and community mapping tools that enable them to fully participate in the research process and exposure characterization, strengthening the community collaborative foundation of CBPR. Environmental justice research, combined with PPGIS-enabled community partners, can inform the translation of evidence-based environmental policies into effective frontline public health practice.

Adoption of a public health exposome approach challenges many of the traditional ways in which health disparities research has been conducted, organized, and funded and will require a paradigm shift for both health disparities researchers and funding agencies. It will require valuing the use of both data driven and hypothesis driven approaches. It will entail the development of new epistemologies and theories for conceptualizing the bio-psychos-social pathways through which environment affects health. It demands a robust bioinformatics infrastructure and taxonomy for linking personal attribute data (genetic, epigenetic, omics, behavior, and psychosocial), direct and indirect environmental exposure data, and health behavior, screening, and outcomes data. It will necessitate the use of new theories, models, methods and analytics to understand and assess those relationships.

Application of an exposure science approach to health disparities has challenged us to rethink how the center was organized to best support translational health disparities research. It has required us to develop new transdisciplinary pedagogies and training programs to prepare investigators in an era of “Big Data”. Embedding a transdisciplinary approach as a required core in future PHE-driven, translational research, for instance, will significantly increase the likelihood of building a sustainable cadre of new generation, environmental public health scientists and health disparities investigators. Rather than creating separate cores around bioinformatics, biostatistics and other research functions we chose to establish an Informatics and Analytics Core that supports the integration of diverse types of data, including biological, tissue, clinical, imaging, and environmental datasets and an expanded range of analytics, including statistical, spatial, and computational analysis. To accomplish this we have had to recruit collaborating investigators with expertise in “Big Data”, mathematical modeling and algorithms, GIS, mapping, predictive modeling and simulation, and other methods supported by a PHE approach.

In summary, these authors offer the PHE as an alternative exposure science paradigm that is particularly relevant for the study of health disparities. The PHE moves away from a traditional, categorical, reductionist approach towards an integrated, systems science approach to understand the causal pathways through which exogenous and endogenous exposures operate, their impact on biological and bio-psycho-social systems, and ultimately, their combined impact on personal health and community-level health disparities.

## Appendix 1. Glossary

*“Big Data”*. Structured and unstructured data sets that are so large and complex that it becomes difficult to process them using personal computers or traditional data processing applications.

*Combinatorial Analysis*. Identifies the number of permutations and combinations in which elements (e.g., data) can be arranged into sets. The elements are usually finite in number, and the arrangement is restricted by certain boundary conditions imposed by the particular problem under investigation.

*Community-Based Participatory Research (CBPR)*. A research approach that facilitates the reciprocal transfer of knowledge, skills, capacity, and power between academic and community partners.

*Community Mapping*. A mapping technology that fosters participatory planning, community education, and cooperative organization.

*Crowdsourcing*. The process of obtaining needed services, ideas, or content by soliciting contributions from a large group of people, especially from an online community.

*Social/Ecological*. Theoretical approach that considers social and ecological systems as resilient, complex, and linked through feedback mechanisms.

*Exogenous*. Developing from external factors.

*Endogenous*. Growing or originating from within an organism.

*Exposome*. The measure of all the exposures of an individual in a lifetime and how those exposures relate to health.

*Geographic Information Systems (GIS)*. A computer system designed to capture, store, manipulate, analyze, manage, and present all types of spatial-temporal and geographical data.

*Graph Theory*. A branch of mathematics concerned about how networks can be encoded and their properties measured.

*Health Disparities*. Inequities that exist when members of certain population groups do not benefit from the same health status as other groups.

*Health Impact Assessment*. A process for assessing the health impacts of policies, plans and projects in diverse communities using quantitative and qualitative data collection methods and community participation.

*Life Cycle Approach*. Considers growth and developmental issues unique to different periods of life.

*Paradigm*. A new theoretical framework for looking at an issue.

*Public Health Exposome Approach*. Uses a systems approach to consider the complex relationships between environmental exposures, personal health outcomes, and population level disparities across the lifespan.

*Public Health Exposome Framework*. Considers the relationships of exogenous and endogenous exposures across multiple levels in four environmental domains: natural, built, social, and policy.

*Public Participatory GIS (PPGIS)*. A specialized application of community-based participatory research that engages community participants in data collection by integrating smart phones, GIS, and web technologies

*Smart Phones*. A mobile phone that combines cellular telephone, Internet access, camera and camcorder, GPS, GIS, accelerometer, light meters and other emerging technologies.

*Spatial-Temporal*. Incorporates qualities of both time and space.

*Systems Theory*. The study of the complex and dynamic relationships and processes through which members of a system act as a whole.

## Appendix 2. PHE Data Repository Data Sources

### Social

1990 U.S. Census Summary Files

2000 U.S. Census Summary Files

2010 U.S. Census Summary Files

ASU GeoDa Center for Geospatial Analysis

Bureau of Labor Statistics Local Area Unemployment Statistics

CMS Community Indicator

DOL/BLS Metropolitan Area Occupational Employment statistics

FIPS (Federal Information Processing Standards) Code

Measures of America of the Social Science Research Council, HD Index and Supplemental Indicators by County and county equivalent, ALL Veterans by county and war

National Center for Education Statistics

Uniform Crime Reporting Program

U.S. Census Current Population Survey

U.S. Census American Fact Finder

U.S. Census American Community Survey

### Built

American Housing Survey

Area Health Resource File

DHUD Affordable Housing-County

DHUD Low Income Housing Tax Credit Property Data

EPA Air Facility System (Aerometric Information Retrieval System-AIRS)

EPA Facility Registry System

Farm Program Atlas data by county

HRSA geospatial Data warehouse

USPO Vacancy Rates (residential and business)

USDA US farmer’s market directory

US DHHS, Center for Medicare & Medicaid Services, Provider of Services File

### Natural

Centers for Disease Control and Prevention, National Environmental Public Health Tracking Network

EPA Air Quality System

EPA Greenhouse gas reporting program

ERS-USDA, State agency data on oil production

EPA-SRSS County level radon

EPA Toxics Release Inventory

EPA Unregulated Contaminant Monitoring Rule (UCMR) program

NASA (Remotely sensed data)

USDA ERS Food Environmental Atlas

USDA Economic Research Service, County Typology codes

US Department of Homeland Security, Federal Emergency Management Agency

USGS Data Gateway

USGS National Water Information System Program

### Policy

Americans for Nonsmokers’ Rights

DOL State Minimum Wage laws

NHTSA States with Primary Seat Belt laws

### Health

Behavioral Risk Factor Surveillance System

CDC Wonder

Center for Medicare & Medicaid Services, Chronic Conditions Database: 2007–2011

Centers for Medicare and Medicaid Community Indicators

Child and Adolescent Health Measurement Initiative, National Survey of Children’s Health

County Health Status Indicators

Dartmouth Atlas of Health Care

Fatality Analysis Reporting System (FARS)

Henry J. Kaiser Family Foundation Life Expectancy at Birth (in years)

Institute for Health Metrics and Evaluation

National Cancer Institute State Cancer profiles

National Center for Health Statistics

National Notifiable Disease Surveillance System

National Vital Statistics System

Surveillance, Epidemiology, and End Results Program (SEER)

University of Wisconsin Population Health Institute County Health Rankings

U.S. Department of Health and Human Services Community Health Status Indicators Report

U.S. Department of Agriculture Supplemental Nutrition program

U.S. Department of Transportation, National Highway Traffic Safety Administration

## Appendix 3. Comparison of Data Analyses Methods

**Table A1:** Comparison of data analyses methods.

Comparitive Information	Multi-Level Modeling	Spatial-Temporal	Combinatorial/Graph Theory
**Type of Data**	Individual and group level data with a small number of variables/factors used to model one (typically) response variable	Geo-coded raster and vector data	Large-scale, heterogeneous, often high-throughput
**Purpose**	To account for the hierarchical and correlation data structure (spatial and temporal), allowing for the simultaneous examination of individual and group-level factors. Can be used for prediction and statistical inference.	To analyze the spatial and temporal relationships among diseases, environments, population characteristics and health disparities within or between defined populations and geographic areas	To detect subtle patterns, latent relationships, and other useful information hidden within vast collections of sometimes-only modest correlations
**Methods**	Mixed model analysis of variance or regression analysis. The units of analysis are usually individuals (at a lower level) nested within contextual/aggregate units (at a higher level). The dependent variable must be examined at the lowest level of analysis.	Uses topological, geometric, or geographic properties of data to generate a geographically weighted regression model of a spatio-temporal phenomenon	Employs graph theoretical algorithms to pinpoint key network structure s and to distill statistically robust inter-related clusters
**Outcomes**	(1) Quantifies the extent to which health outcomes are clustered by neighborhood and community grouping; (2) quantifies how individual risk factors vary from neighborhood to neighborhood; and (3) quantifies the relative importance of individual, neighborhood and societal level exposures in predicting individual health outcomes.	Can be used to examine the relationships and changes in patterns over time of environmental hazards, socioeconomic status, socially vulnerable neighborhoods, and health disparities.	Analyzes the entire search space, reduces dimensionality to manageable levels, and generates hypotheses suitable for testing with orthogonal methods
**Strengths**	Using contextual factors beyond individual factors allows for a more accurate identification of at-risk populations, which can be useful when planning health programs	Providing information on spatial and temporal relationships among variables.	Unbiased and immune to preconception, scalable to datasets of immense size, exploits novel mathematical techniques to overcome combinatorial bottlenecks
**Limitations**	Group-level correlations can be mistakenlyattributed to individual-level causes, since between-studyvariation is typically observational even when individual studies arerandomized experiments	Spatial dependency leads to spatial autocorrelation which violates standard statistical techniques that assume independence among observations.	Sufficient data needed to compute correlation structures, requires special knowledge for implementation, tuning and refinement
